# Upregulated UCA1 contributes to oxaliplatin resistance of hepatocellular carcinoma through inhibition of miR‐138‐5p and activation of AKT/mTOR signaling pathway

**DOI:** 10.1002/prp2.720

**Published:** 2021-02-10

**Authors:** Guolin Huang, Li Li, Chaoyong Liang, Fei Yu, Cuifang Teng, Yingxing Pang, Tongtong Wei, Jinjing Song, Hanlin Wang, Xiaoli Liao, Yongqiang Li, Jie Yang

**Affiliations:** ^1^ Department of Pharmacology School of Pharmacy Guangxi Medical University Nanning Guangxi People’s Republic of China; ^2^ Department of Chemotherapy Affiliated Cancer Hospital Guangxi Medical University Nanning Guangxi People’s Republic of China; ^3^ Department of Internal Medicine First Affiliated Hospital Guangxi Medical University Nanning Guangxi People’s Republic of China

**Keywords:** hepatocellular carcinoma, long noncoding RNA, miR‐138‐5p, oxaliplatin resistance, UCA1

## Abstract

Hepatocellular carcinoma (HCC) inevitably developed oxaliplatin (OXA) resistance after long‐term treatment, but the mechanism remains unclear. Here, we found that LncRNA UCA1 was upregulated in most of OXA‐resistant HCC tissues and cells (HepG2/OXA and SMMC‐7721/OXA). Follow‐up analysis and online Kaplan–Meier Plotter revealed that HCC patients with high UCA1 level had a shorter survival compared with those with low expression. Overexpression of UCA1 increased OXA IC50 in HepG2 and SMMC‐7721 cells, whereas knockdown of UCA1 decreased OXA IC50 in resistant counterparts. Moreover, dual luciferase reporter assay showed that co‐transfection of UCA1‐WT plasmid with miR‐138‐5p mimics enhanced fluorescence signals, whereas co‐transfection of UCA1‐Mut plasmid and miR‐138‐5p mimics did not induce any changes. Consistently, UCA1 levels in HepG2/OXA and SMMC‐7721/OXA cells were downregulated after transfected with miR‐138‐5p mimics. UCA1 silencing or transfection of miR‐138‐5p mmics inhibited the activation of AKT and mTOR in HepG2/OXA and SMMC‐7721/OXA cells, whereas UCA1 overexpression increased the phosphorylated AKT and mTOR levels in parental counterparts. Rapamycin or miR‐138‐5p mimics similarly suppressed the activation of AKT and mTOR, whereas UCA1 overexpression exert opposite roles. Interestingly, administration of rapamycin or miR‐138‐5p mimics apparently antagonized the effects of UCA1 on AKT and mTOR activation. Besides, depletion of UCA1 triggered more dramatic regression of HepG2 xenografts than that of HepG2/OXA xenografts with OXA treatment and impaired the p‐AKT and p‐mTOR levels in vivo. In conclusion, our findings provide the evidence that UCA1 may contribute to OXA resistance via miR‐138‐5p‐mediated AK /mTOR activation, suggesting that UCA1 is a potential therapeutic target for HCC.

## 1 INTRODUCTION

Liver cancer is the fourth leading cause of global mortality, and approximately 80% liver cancers are hepatocellular carcinoma (HCC).^1^, ^2^ The incidence of HCC in China is higher than that in other countries and the number of new cases and deaths in China account for nearly half^3^, ^4^ Guangxi province is a high‐risk area for HCC in China where the mortality of HCC reaches 40%, mainly due to hepatitis B virus (HBV) infection, aflatoxin intake, and polluted drinking water.^5^, ^6^, ^7^ HCC is always known as one of the most aggressive cancers that often progressing silently. Therefore, most HCC patients (∼80%) usually present with advanced stage and lose the chance of surgical resection, liver transplantation, or localized tumor ablation.^8^ Systemic chemotherapy is commonly used in advanced HCC as a palliative therapy in order to relieve symptoms and improve quality of life, control tumor growth or spread and prolong overall survival (OS).^9^, ^10^ The first approved systemic agent to demonstrate a survival benefit in patients with advanced HCC was sorafenib, a multikinase inhibitor that inhibits tumor angiogenesis and growth.^11^ Oxaliplatin (OXA) is a third‐generation platinum agent with obvious advantages, including great tolerance, broad therapeutic window, as well as more cost‐effectiveness than sorafenib.^12^ OXA‐containing regimens such FOLFOX4 (OXA plus infusional‐fluorouracil (FU) and leucovorin (LV) (FOLFOX4)), XELOX (capecitabine plus OXA), and GEMOX (Gemcitabin plus OXA) have shown improved clinical activity against HCC.^13^ FOLFOX4 was included in Chinese national clinical practice guidelines for HCC^14^ and approved for the systemic treatment of locally advanced or metastatic HCC in China in 2013.^14^, ^15^ However, despite initial sensitivity to oxaliplatin, most HCC eventually develop OXA resistance which leads to treatment failure.^16^ Therefore, OXA resistance has become a bottleneck that limits the clinical efficacy of HCC therapy.

Long noncoding RNAs (lncRNAs) are a class of heterogeneous nonprotein‐coding RNAs with lengths ranging from 200 to 100,000 nt. lncRNAs regulate gene expression at multiple levels, including alternative splicing, chromatin modification, as well as protein localization and activity, due to their ability to bind to DNA, RNAs, and proteins.^17^ Recently, lncRNAs have been discovered to play critical roles in the regulation of tumor proliferation, invasion, migration, and chemo‐resistance.^18^ Particularly, upregulated lncRNAs seem to exhibit tumor‐promoting roles, whereas downregulated lncRNAs possess tumor‐suppressive abilities.^19^ For instance, lncRNA HOTAIR and MALAT‐1 were upregulated in most HCC patient tissues, whereas inhibiting their expression could sensitize HCC cells to doxorubicin and cisplatin, respectively.^20^, ^21^ Conversely, L Wu et al. found that the decreased lncRNA KRAL is closely related to the resistance to 5‐fluorouracil in HCC.^22^ UCA1 (urothelial carcinoma associated 1) is a lncRNA that was firstly identified in human bladder carcinoma,^23^ whose expression is also found to be elevated in many other cancers.^19^, ^24^, ^25^, ^26^ UCA1 has been shown to facilitate the cancer cell growth, migration, invasion, metastasis and drug resistance by activating PI3K/AKT, mTOR/STAT3, and other signaling pathways.^27^, ^28^ Moreover, UCA1 induced multidrug resistance to cisplatin and gemcitabine in bladder cancer cell by activating the transcription factor CREB after declining the expression of miR‐196a‐5p.^18^ Silencing UCA1 upregulated cleaved PARP protein expression and inhibited the antiapoptosis protein Bcl‐2, resulting in enhanced apoptosis and depressed chemotherapy resistance to adriamycin in gastric cancer cells.^29^ Besides, UCA1 activated AKT/mTOR signaling, thus promoting NSCLC cells to undergo epithlial‐mesenchymal transition (EMT) against Gefitinib and Erlotinib therapies.^30^ However, the role of UCA1 in oxaliplatin resistance of HCC is still poorly understand.

In this study, we found that UCA1 was upregulated in OXA‐resistant HCC specimens and cells, and was closely associated with a shorter survival in HCC patients. Furthermore, miR‐138‐5p was shown to act as a potential target miRNA of UCA1 through bioinformatic analysis, dual‐luciferase reporter assay, and qRT‐PCR. In addition, overexpression of UCA1 induced the activation of AKT and mTOR and this effect was similarly inhibited by either administration of rapamycin or upregulation of miR‐138‐5p. In vivo model consistently showed that high UCA1 level maintained the growth of OXA‐resistant exonegraft as well as elevated p‐AKT and p‐mTOR, which could be antagonized by UCA1 depletion. Together, our findings suggest that UCA1 may facilitate OXA resistance of HCC by miR‐138‐5p‐mediated the activation of the AKT/mTOR signaling and act as a potential therapeutic target for HCC.

## 2 MATERIALS AND METHODS

### 2.1 Information of clinical sample and enrollment criteria

Electronic medical records from 2011 to 2017 were retrospectively studied to screen out 75 appropriate candidates as following criteria: (a) Written informed consents were obtained from patients. (b) Age exceeded 18 years old. (c) ESCO scored no more than 2. (d) Patients were diagnosed with primary HCC according to "Guidelines for Diagnosis and Treatment of Primary Liver Cancer in China (2017 Edition)".^14^ (e) Surgical resection of HCC had been done and resected specimens were available. (f) No chemotherapy or radiotherapy prior to surgery; (g) Tumor recurrence after OXA chemotherapy. (h) No other anticancer drugs prior to OXA chemotherapy.^31^ According to the standard of RECIST,^32^ a total enrollment of 75 patients was classified as 36 OXA‐sensitive cases and 39 OXA‐insensitive cases by their clinical efficacy score. The clinicopathological information was obtained from medical records and is described in supplementary Table S3. The follow‐up of 70 enrollments was traced including survival time, healthy condition, and occurrence of any complication. The analysis of Kaplan–Meier curve was performed based on follow‐up information and online database Kaplan–Meier Plotter which including the data from GEO, EGA, and TCGA (http://kmplot.com/analysis/index.php?p=background). This study was agreed by the Ethics Committee of Guangxi Medical University (IRB NO.2018‐824).

### 2.2 Culture of cell lines and establishment of oxaliplatin‐resistant cell lines

Human HCC cell lines (HepG2 and SMMC‐7721) were purchased from the Chinese Academy of Sciences Cell Bank (China, Shanghai), and normal liver cell line HL‐7702 was obtained from American Type Culture Collection (ATCC). Cells were cultured in Dulbecco's Modified Eagle Medium (DMEM) containing 10% fetal bovine serum (Gibco) and 1% (v/v) antibiotics (100 U/ml penicillin and 100 μg/ml streptomycin) at 37 °C, 5% CO_2_. Two OXA‐resistant HCC cell lines HepG2/OXA and SMMC‐7721/OXA were established in vitro by continuous exposure to gradually increased concentration of OXA from 0.01 to 5.0 μΜ for at least 6 months as reported previously.^33^

### 2.3 Lentivirus and plasmid transfection

HCC parental and their resistant cells were seeded into 6‐well plates and incubated overnight. HepG2/OXA and SMMC‐7721/OXA cells were transfected with 1 × 10^7^ TU/ml of LV‐shUCA1 or a negative control LV‐shNC, HepG2, and SMMC‐7721 cells were transfected with 1 × 10^7^ TU/ml of LV‐UCA1 or a negative control LV‐NC (GenePharma, Shanghai, China), respectively. After 24 hours incubation, the transfection efficiency was detected by fluorescence microscope (BioTek, USA). Cells were selected by 3 μg/ml of puromycin (Sorabol, China) for 2 weeks and then the cells were harvested for qRT‐PCR analysis. The target sequence for LV‐shUCA1 was as follows: sense strand, 5′‐GGGTTCACCATTCCAGAATAA‐3′, antisense strand, 3‘‐GGGTTCACCATTCCAGAATAA‐5′. Besides, 2.5 μg of miRNA‐138‐5p mimics or NC (GenePharma, Shanghai, China) were transiently transfected into HepG2/OXA and SMMC‐7721/OXA cells, respectively, using Lipofectamine 3000 (Invitrogen) according to the manufacturer's instructions. These cells were applied for related experiments after 72 hours transfection.

### 2.4 Methyl thiazolyl tetrazolium (MTT) assay for detecting IC_50_

As we previously reported,^34^ cells were seeded into 96‐well plates at 3000 cells/well in triplicate and incubated overnight. Cells were treated with a series of concentration of OXA (0 μmol/L, 5 μmol/L, 10 μmol/L, 20 μmol/L, 40 μmol/L, and 80 μmol/L) for 48 hours and then 20 μl of MTT reagent (0.5 mg/L) (Sigma, Schnelldorf, Germany) was added to each well, and incubated the plates at 37°C for 4 hours. The supernatant was removed and 150 μl of DMSO was added to each well. After incubation for 4 hours min at 37°C, the optical density (OD) of each wells was measured by absorbance at 490 nm in a microplate reader (BioTek, USA). The OD of treated cells were normalized to the OD of nontreated cells. Each experiment was repeated three times. Cytotoxicity curves were obtained using Graphpad software by plotting the measured cell viability (%) by MTT assay against OXA concentrations.

### 2.5 Colony formation assay

HCC cells (300 cells/well) were plated into 6‐well plate in triplicate and maintained in complete DMEM with or without OXA with the indicated concentraion (5.45 μmol/L for HepG2 and HepG2/OXA, 6.38 μmol/L for SMMC‐7721 and SMMC‐7721/OXA) for 2 weeks until the macroscopic colonies formed. Colonies were fixed with 4% paraformaldehyde for 15 minutes and then stained with 1% crystal violet for 5 minutes. Wells were washed with H2O to remove residue crystal violet. A colony with more than 50 cells was defined as a colony and the number of colonies per well were counted. Colony‐forming efficiency (%) = (number of colonies / number of inoculated cells) * 100%.^35^

### 2.6 Dual‐luciferase reporter assay

Bioinformatics online softwares, DIANA tools lncBase Predicted v.2 (https://carolina.imis.athena‐innovation.gr/diana_tools/web/index.php?r=lncbasev2%2Findex‐predicted) and RNA22 version 2.0 (https://cm.jefferson.edu/rna22/), were used for predicting the potential targets of UCA1. The results suggested that miR‐138‐5p would be able to targeted by UCA1. Therefore, the confirmation of the combination between UCA1 and miR‐138‐5p was conducted by using the dual‐luciferase reporter assay system (Promega, USA). The miR‐138‐5p binding sites in UCA1 sequences (wild‐type or mutant type) were embedded into the luciferase reporter vector pSI‐check2‐h‐lncRNA‐UCA1‐WT or pSI‐check2‐h‐lncRNA‐UCA1‐Mut, respectively (Hanheng Biotechnology Co., Shanghai, China). 293T cells grown in the 96‐well plate were co‐transfected with either miR‐138‐5p mimics or miR‐NC and luciferase reporter comprising wild type (UCA1‐WT) or mutant UCA1 (UCA1‐Mut 1 + 2) fragment, using Lipofectamie 3000 (Invitrogen). Cells were harvested 48 hours after transfection and luciferase activity was detected as chemiluminescence in a luminometer (Carl Zeiss) according to the manufacturer's protocol.

### 2.7 RNA extraction and qRT‐PCR assays

Total RNA was extracted from HCC tissues and cells using RNAiso Plus (TAKARA, Japan). PrimeScript™ RT reagent Kit with gDNA Eraser (TAKARA, Japan) was used to synthesize cDNA. RT‐PCR was performed using SYBR Premix Ex Taq II kit (Takara, Dalian, China) and ABI 7500 PRISM 7500 (Applied Biosystems). The RT‐PCR reaction conditions were as follows: stage 1, 95°C for 10 minutes; stage 2, 40 cycles of 95°C for 5 seconds, 60°C for 34 seconds. Relative expression was normalized to GAPDH or U6 expression and calculated with the 2^–ΔΔCt^ method.^36^ The experiments were set in triplicates. Primers were listed in supplementary Table S1.

### 2.8 Western blot

Western blot was performed as previously reported.^37^ Whole cells or tissues extracts were prepared using 200ul RIPA lysis buffer. Then 30 μg of each isolated protein was separated by 10% SDS‐PAGE gel and transferred onto NC membrane. The whole blot membrane then was blocked using 5% skim milk. Membrane was probed with respective primary antibodies which are listed in supplementary Table S2. at 4°C overnight. Secondary antibody anti‐rabbit IgG (1:30000, DyLight™ 800 4X PEG Conjugate, CST, US) or anti‐mouse IgG (1:10000, Licor, USA) were used for visualization. Protein bands were then imaged using the infrared fluorescence imaging system Odyssey (LI‐COR, USA). The intensity of each band was calculated using ImagJ Plus. The western blot assays were conducted at least three times.

### 2.9 Animal model

All procedures for animal experiments were performed in the instruction of Guideline for ethical review of animal welfare (GB/T35892‐2018), and were approved by the Experimental Animal Welfare and Ethics Committee of Guangxi Medical University. The athymic BALB/c male nude mice aged 4‐5 weeks old and weighted about 14–16 g were used for establishing model of xenotransplanted tumors. For the subcutaneous xenograft model, HepG2/OXA‐shUCA1 and HepG2/OXA‐shNC cells were subcutaneously injected at a density of 1 × 10^7^ cells/ml into the flanks of mice (200 µl/mice,10 mice/group). After implantation of tumor cells, tumor length (L) and width (W) were measured twice a week and tumor volume (V) was calculated using a formula: V(mm^3^) = (L × W^2^)/2.^38^ The mice were further randomized to two treatment subgroups of either intraperitoneal injection of 10 mg/kg oxaliplatin or the same volume of saline once a week for 4 weeks. When the largest length of tumor exceed 20 mm, mice were sacrificed and the tumor was collected, weighted, and stored in ultra‐low temperature freezer for further use.

### 2.10 In situ hybridization (ISH) of UCA1

UCA1 expression in HCC tissues was measured using UCA1 ISH kit according to the manufacturer's protocol. All tissue sections were fixed by paraffin and then were deparaffinized with xylene and 100% ethanol. Subsequently, sections were rinsed in phosphate‐buffered saline (PBS), fixed in 1% paraformaldehyde for 10 minutes at 25°C, then incubated in hybridization buffer for 4 hours at 42°C. The hybridization was performed using digoxin‐labeled probe of UCA1 in the thermostat for reaction at 42℃overnight. After RNA in situ hybridization, we used an mouse anti‐digoxin horseradish peroxidase (HRP)‐conjugate and incubated at 37°C for 60 minutes followed by diaminobenzidine tetrahydrochloride (DAB) and hematoxylin staining. Finally, all images were obtained using a TS100 ‐F (Nikon, Japan) confocal microscope. A semi‐quantitative score for ISH was calculated by multiplying the percentage of positive cells (P: 0~5% = 0,6~25% = 1, 26~50% = 2, >50% = 3) and the intensity (I: negative = 0, weak = 1, moderate = 2, strong = 3). Formula: Q = P × I.^39^ The final scores were divided into two levels: UCA1 low expression (0~3) and UCA1 high expression (3~9).

### 2.11 Statistic analysis

IBM SPSS Statistics 19.0 software was applied for analyzing data which were displayed as mean ± standard deviation (SD) and assessed by one‐way analysis of variance analysis. LSD test was used for comparison between intragroups and χ^2^ analysis was conducted for analyzing the expression of UCA1 in ISH results and the relationship of clinicopathological characteristics. Independent prognostic factors were evaluated by univariate and multivariable Cox proportional hazards regression analysis. A *p* value <0.05 was considered statistically significant.

## 3 RESULTS

### 3.1 UCA1 is upregulated in OXA‐resistant HCC clinical samples and predicted poor prognosis

**FIGURE 1:**
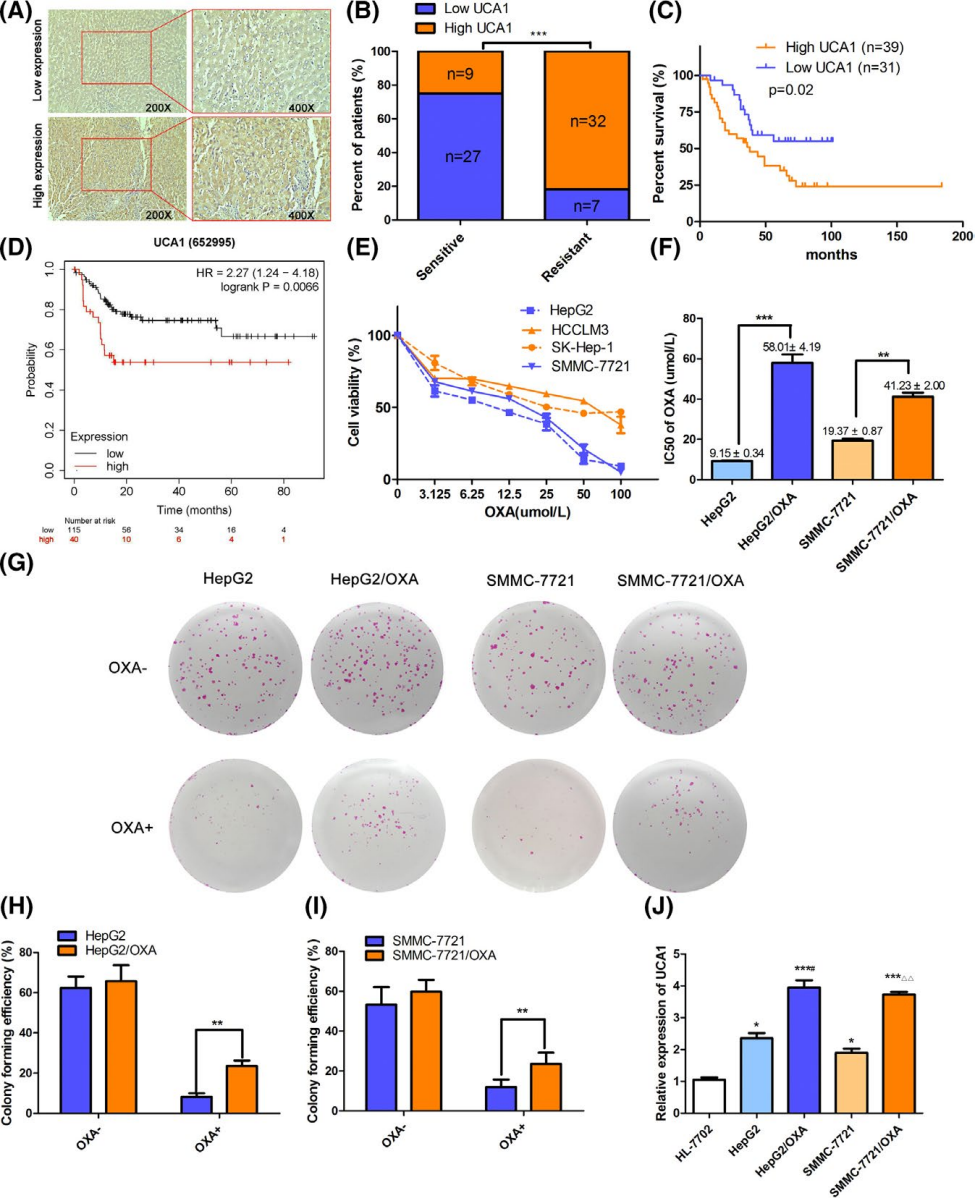
UCA1 is preferentially upregulated in OXA‐resistant HCC patient tissues and cells (A), Representative images ofin situ hybridization showing low or high expressions of UCA1within human HCC tissues (Original magnification, 200× or 400×). (B) Relative density of UCA1 signal in OXA‐resistant or OXA‐sensitive HCC specimens was quantified by Image J, and the significant was determined by student's *t* test. (C) Kaplan‐ Meier curves for OSin HCC patients with low or high expression of UCA1 (n = 70, *p* = 0.02, log‐rank test). (D) Kaplan‐Meier curve for OS in Asian HCC patients with low or high expression of UCA1(n = 155, *p* = 0.0066). (E) Cell survival rates were measured by MTT in four HCC cell lines treated with OXA at the indicated concentrations (0 μmol/L to 100 μmol/L) for 48 h.Graphs are mean ±SD for 3 independent experiments. F, MTT assays indicate that IC50 value of OXA is largely increased in resistantHepG2/OXA and SMMC‐7721/OXA cells. ***p < 0.001 or **p < 0.01 compared to their parental cells HepG2 or SMMC‐7721.G‐I, Colony formation assay was used to determine the colony forming efficiency of the indicated HCC cells with or without OXA treatment. Bars are presented as the mean ± SD from 3 independent experiments. **p < 0.01 compared to their parental cells. J, Relative expression of UCA1 in normal liver cells HL‐7702, HepG2 and SMMC‐7721 and their resistant HepG2/OXA and SMMC‐7721/OXA cells was detected by qRT‐PCR. Data is expressed as mean± SD from 3 independent experiments. *p < 0.05, **p < 0.01, ***p < 0.001, compared to HL‐7702; #*p* < 0.05, compared to HepG2; △△*p* < 0.01, compared to SMMC‐7721.

Previous research has illustrated that UCA1 positively regulates chemo‐resistance in several types of cancers. Therefore, we assumed that the expression of UCA1 might increase in OXA‐resistant HCC tissues and cells. To explore its function in HCC, we first analyzed the expression of UCA1 in HCC tissues by ISH assay. Total 75 recruited patients were classified into OXA‐sensitive group and OXA‐insensitive group as mentioned in methods. Thirty‐six individuals were assigned to OXA‐sensitive group, whereas the rest of 39 cases belonged to OXA‐insensitive group. As the results of ISH shown in Figure 1A,B, high expression of UCA1 appeared in 82% of OXA‐resistant individuals, whereas only 25% of OXA‐sensitive tissues showed high expression of UCA1 (χ^2^ = 24.586, *p* < 0.001). Next, the relationship between UCA1 expression and clinicopathological characteristics of HCC patients was analyzed as shown in supplementary Table S3. The results illustrated that the high expression of UCA1 positively correlated with serum AFP (χ^2^ = 3.921, *p *< 0.05), tumor size (χ^2^ = 5.591, *p *< 0.05) and distant metastasis (χ^2^ = 5.345, *p *< 0.05). According to the follow‐up records of 70 patients, a smaller proportion of HCC patients with high expression of UCA1 survived compared to those with low UCA1 expression (*p *<* *0.05, Figure 1C). Moreover, online Kaplan–Meier Plotter created from the public databases including GEO, EGA, and TCGA ^40^ showed that Asian HCC patients with high expression of UCA1 had a shorter survival time compared to those with low UCA1 expression (*p *< 0.01, Figure 1D). To determine whether UCA1 is prognostically independent of the clinical variables it correlates with in Table S3, we also performed the univariate and multivariate Cox proportional hazards analysis. As shown in Table S4, the univariate analysis showed that overall survival (OS) was significantly lower in HCC patients with high expression of UCA1 (*p* = 0.021), or HBV infection (*p* = 0.009), or Child‐Pugh classification C (*p* = 0.019). However, multivariate Cox proportional hazards analysis revealed that none of the three factors was an independent prognostic factor for OS in HCC patients. Collectively, the clinical evidence suggests that the high expression of UCA1 might be involved in progression and/or oxaliplatin resistance in HCC.

### 3.2 UCA1 expression is elevated in HCC cells resisted to OXA

To generate an in vitro cellular model of OXA resistance, we firstly tested the OXA efficacy on HCC cell lines HepG2, SMMC‐7721, HCC‐LM3, and SK‐HEP‐1. Figure 1E showed that the cell survival rates were lower in HepG2 and SMMC‐7721 cells than that in HCC‐LM3 and SK‐HEP‐1 cells at the concentration from 0 μmol/L to 100 μmol/L of oxaliplatin, indicating that HepG2 and SMMC‐7721 cells are more sensitive to OXA treatment than HCC‐LM3 and SK‐HEP‐1 cells. Therefore, two OXA‐resistant cell lines HepG2/OXA and SMMC‐7721/OXA were established from HepG2 and SMMC‐7721 cells by continuous exposure to gradually increased concentration of OXA. Compare to their parental cells, the IC_50_ value of HepG2/OXA or SMMC‐7721/OXA was 58.01 ± 4.19 μmol/L (vs 9.15 ± 0.34 μmol/L, *p *< 0.001), or 41.23 ± 2.00 μmol/L (*vs* 19.37 ± 0.87 μmol/L, *p *<* *0.01), respectively (Figure 1F). The resistance index (RI, RI = IC_50_ of drug‑resistant cells/IC_50_ of parental cells) ^1^ of HepG2/OXA and SMMC‐7721/OXA was 6.34 and 2.13 separately. Additionally, colony formation assay also proved that colony‐forming efficiency under OXA treatment was apparently enhanced in HepG2/OXA and SMMC‐7721/OXA compared with that in their parental cells, but no significant difference between HepG2/OXA and SMMC‐7721/OXA and their parental cells without OXA treatment (*p *<* *0.01) (Figure 1G‐I). Furthermore, Figure 1J demonstrated the expression of UCA1 was higher in two parental cells (HepG2 and SMMC‐7721) compared with normal liver cells (HL‐7702) (*p *<* *0.05), and the expression of UCA1 was further higher in two OXA‐resistant cells (HepG2/OXA and SMMC‐7721/OXA) compared with their parental counterparts (*p *<* *0.001), suggesting the potential involvement of UCA1 in OXA resistance of HCC.

### 3.3 UCA1 induces OXA resistance in HCC cells

**FIGURE 2:**
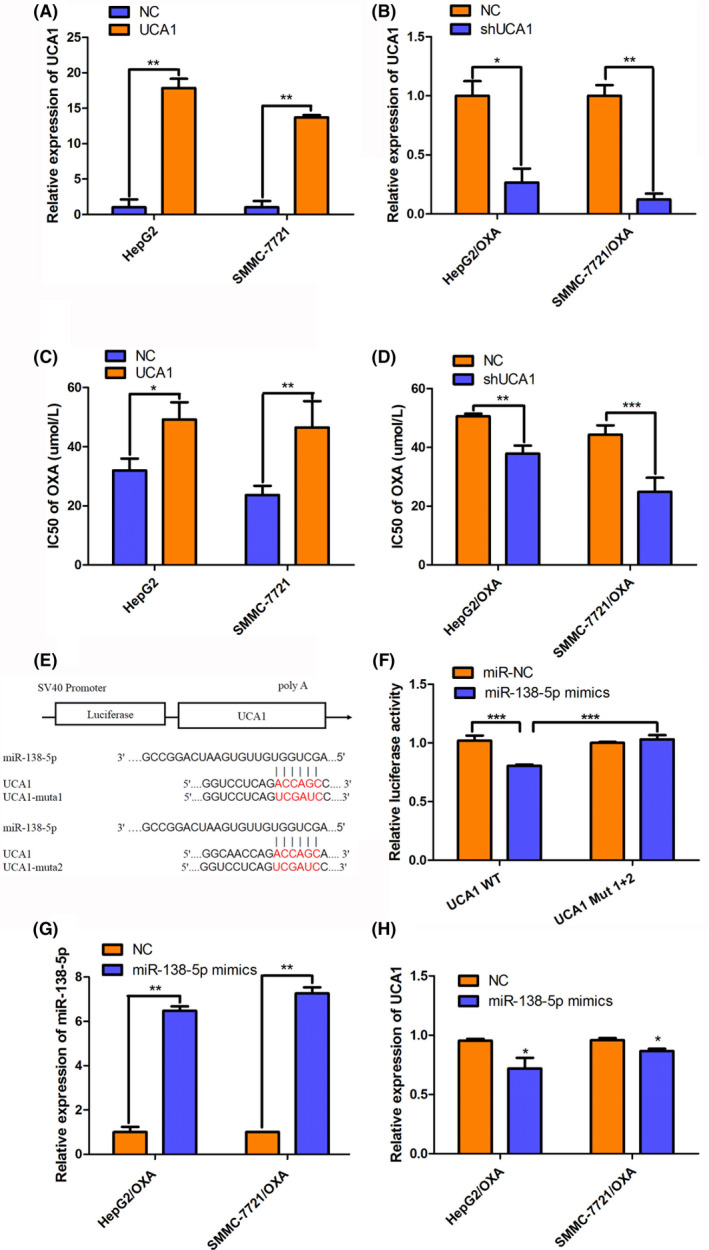
UCA1 induces OXA resistance in HCC cells and contains the binding sites of miR‐138‐5p. (A‐B) Expression of UCA1 was measured using qRT‐PCR in HepG2 and SMMC‐7721 cells transfected with UCA1 or NC lentivirus (A), or in HepG2/OXA and SMMC‐7721/OXA cells transfected with shUCA1 or shNC lentivirus (B). (C‐D) The IC50 value of OXA was detected by MTT in the cells above. E, Schematic diagram illustrated the complementary bound within miR‐138‐5p and UCA1 3’‐UTR with binding sites predicted by bioinformatics tools (DIANA tools lncBase Predicted v.2, https://carolina.imis.athenainnovation. gr/diana_tools/web/index.php?r = lncbasev2%2Findex‐predictedand RNA22 version 2.0, https://cm.jefferson.edu/rna22/). F, Dual luciferase gene assay was conducted in HEK‐293T cells cotransfected with miR‐138‐5p mimics or NC and UCA1 wild type (UCA1 WT) or UCA1 mutant type (UCA1 Mut 1 + 2). G‐H, Relative expression of miR‐138‐5p or UCA1 in HepG2/OXA and SMMC‐7721/OXA cells after transfection with miR‐138‐5p mimics. All Data are presented as the mean ± SD from 3 independent experiments. *p < 0.05, **p < 0.01, ***p < 0.001, compared to NC groups.

Since the enhanced expression of UCA1 was found in HepG2/OXA and SMMC‐7721/OXA, we hypothesized and tested that the elevated UCA1 could induce OXA resistance in HCC cells. As expected, the expression of UCA1 had 17.83‐fold or 13.70‐fold increase in UCA1‐overexpressed HepG2 and SMMC‐7721 cells compared with their negative control (NC) counterparts, respectively. Concomitantly, IC_50_ value of OXA was dramatically increased after successfully overexpression of UCA1 in parental HepG2 and SMMC‐7721 cells (Figure 2A,C). On the other hand, after UCA1 knockdown by transfection of UCA1 interference lentivirus, the expression of UCA1 declined 73% and 88% and IC_50_ value of OXA decreased obviously in HepG2/OXA‐shUCA1 and 7721/OXA‐shUCA1 cells compared with their NC groups (Figure 2B,D). Together, these results suggest that UCA1 contributes to OXA resistance and UCA1 knockdown enhances the sensitivity to OXA in HCC cells.

### 3.4 UCA1 contains the binding sites of its inhibitor miR‐138‐5p

Next, we applied the bioinformatics prediction software DIANA tools lncBase Predicted v.2 and RNA22 version 2.0 to predict the potential targets of UCA1. The results suggested that miR‐138‐5p would be able to targeted by UCA1. Then, we generated mutations in UCA1 to explore whether miR‐138‐5p could potentially bind to the sequence of 3’‐terminis” ‐ACCAGC‐” of UCA1 (Figure 2E). The results of dual‐luciferase reporter assay showed that co‐transfection of UCA1‐WT plasmid with miR‐138‐5p mimics caused a significant decrease in fluorescence signals compared to the group with co‐transfection of UCA1‐WT plasmid and miR‐138‐5p NC (0.8 ± 0.02 vs 1 ± 0.08, *p *=* *0.000937), whereas co‐transfection of UCA1‐Mut plasmid with two point mutations in binding sites and miR‐138‐5p mimics did not induce any significant change in fluorescencse signals compared to the group with co‐transfection of UCA1‐Mut plasmid and miR‐138‐5p NC (1.03 ± 0.06 vs 1 ± 0.01, *p* > 0.05) (Figure 2E‐F). These data suggest that UCA1 could be regulated by miR‐138‐5p. Additionally, we identified what happens to endogenous UCA1 when the miRNA mimic is transfected into the normal or resistant cells, separately to the DLR assay. We, respectively, transfected either miR‐138‐5p mimics or their NC mimics into HepG2/OXA and 7721/OXA cells which highly express endogenous UCA1. As shown in Figure 2G,H, qRT‐PCR assays revealed that miR‐138‐5p levels in HepG2/OXA and 7721/OXA cells were greatly elevated after transfection of miR‐138‐5p mimics compared with their NC groups (1.01 ± 0.23 vs 6.48 ± 0.20, *p* = 0.0078; 0.98 ± 0.01 vs 7.26 ± 0.28, *p* = 0.0032, Figure 2G). Furthermore, UCA1 levels in HepG2/OXA and 7721/OXA cells were significantly downregulated after transfected with miR‐138‐5p mimics compared with their NC groups (0.95 ± 0.03 vs 0.72 ± 0.20, *p* = 0.043; 0.96 ± 0.03 vs 0.87 ± 0.04, *p* = 0.011, Figure 2H). These data are in consistent with the result of DLR assay and further supports that miR‐138‐5p does in fact regulate UCA1 endogenously in HCC cells.

### 3.5 UCA1 induces OXA resistance through suppression of miR‐138‐5p expression and activation of AKT/mTOR pathway

**FIGURE 3:**
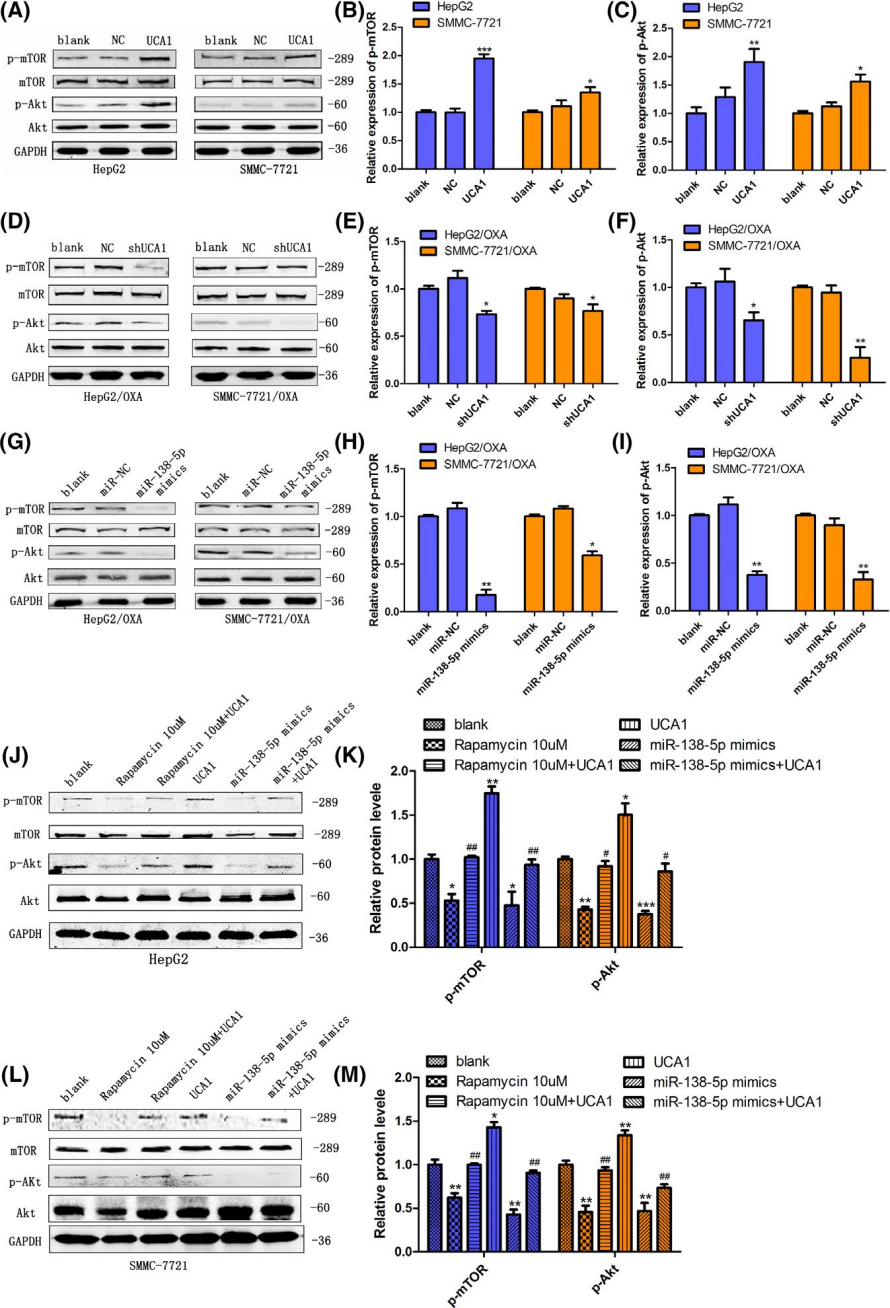
UCA1 promotes OXA resistance through inhibition of miR‐138‐5p expression and activation of AKT/mTOR pathway. (A‐C) Western blot analysis was performed to detect the levels of AKT, mTOR and their phosphorylated forms after overexpression of UCA1 in HepG2 and SMMC‐7721 cells, or knockdown of UCA1 in HepG2/OXA and SMMC‐7721/OXA cells (D‐F), respectively. (G‐I) Western blot analysis demonstrated that overexpression of miR‐138‐5p in HepG2/OXA and SMMC‐7721/OXA cells by transfection with mimics leads to inhibition of p‐AKT and p‐mTOR. Bars are mean ± SD from 3 independent experiments. *p < 0.05, **p < 0.01, ***p < 0.001, compared to blank groups. J‐M, Western blot analysis was used to measure the levels of AKT, mTOR and their phosphorylated forms in HepG2 and SMMC‐7721 cells treated with AKT/mTOR inhibitor rapamycin (10 μmol/L, 24 h), or overexpressed UCA1, or transfected with miR‐138‐5p mimics, or combined administration of rapamycin or miR‐138‐5p mimics with UCA1. Bars are mean ± SD from 3 independent experiments. *p < 0.05, **p < 0.01, ***p < 0.001, compared to blank groups; # *p* < 0.05, ## *p* < 0.01, compared to overexpressing‐UCA1 groups

We previously proved that the activation of AKT/mTOR axis paly a predominant role in invasion and migration of HCC by inducing EMT transformation.^37^ Thus, we were interested to dissect whether AKT/mTOR signaling might be involved in the regulatory role of UCA1 in OXA resistance of HCC cells. The results of western blot illustrated that the expression of p‐AKT and p‐mTOR was obviously increased (*p *<* *0.001) in both HepG2 and SMMC‐7721 cells with UCA1 overexpression (Figure 3A‐C). Conversely, knockdown of UCA1 expression in HepG2/OXA and SMMC‐7721/OXA cells significantly decreased the expression of p‐AKT and p‐mTOR (Figure 3D‐F). Interestingly, miR‐138‐5p was assumed as a transcriptional suppressor of UCA1.^41^ Thereby, we explored whether upregulation of miR‐138‐5p expression could cause the opposite effect on AKT/mTOR pathway activation induced by high UCA1 expression. As expected, we found that both UCA1 expression (Figure 2H) and the level of phosphorylated AKT and mTOR in HepG2/OXA and SMMC‐7721/OXA cells were greatly descended after transfection with miR‐138‐5p mimics (*p *<* *0.01 or *p *<* *0.05) (Figure 3G‐I). Furthermore, we used mTOR/AKT inhibitors in conjunction with UCA1 overexpression and/or miR‐138‐5p mimics to determine if the effect of UCA1 overexpression is mediated by miR‐138‐5p directly. As PI3 K inhibitor LY294002 has been reported to display its effect in low significance in HCC cells, whereas mTOR inhibitor rapamycin was found to robustly inhibit the activation of mTOR and AKT ^42^, ^43^ and was more effective than LY294002 in HCC cells.^44^ Thus, we used rapamycin as mTOR/AKT inhibitor to perform validation experiments. We found that either treatment with rapamycin (10 uM, 24 h) or transfection of miR‐138‐5p mimics in HepG2 and SMMC‐7721 cells similarly suppressed the activation of mTOR and AKT as represented by reduced AKT and mTOR phosphorylation, whereas overexpression of UCA1 enhanced p‐mTOR and p‐AKT activities in these cells (Figure 3J‐M). Interestingly, administration of rapamycin or miR‐138‐5p mimics apparently antagonized the facilitating effects of UCA1 on the activation of mTOR and AKT to a similar extent in HepG2 and SMMC‐7721 cells (Figure 3J‐M). Taken together, our results indicate that the effect of UCA1 overexpression on the activation of mTOR and AKT may be mediated by miR‐138‐5p.

### 3.6 Knockdown of UCA1 re‐sensitizes HCC cells to OXA treatment partially through inactivation of AKT/mTOR pathway in vivo

**FIGURE 4:**
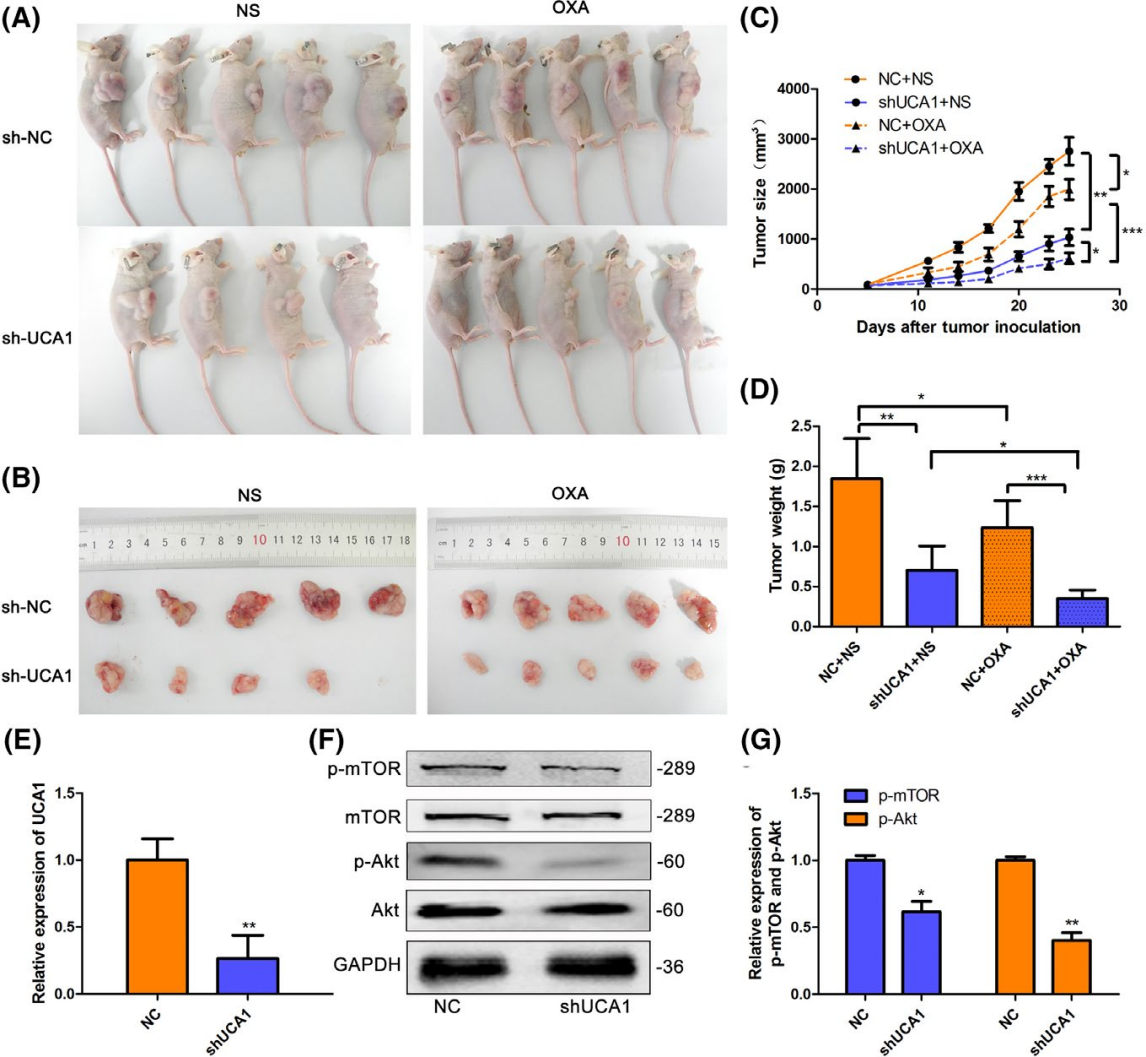
Depletion of UCA1 re‐sensitizes HCC cells to OXA treatment partially through inactivation of AKT/mTOR pathway in vivo. (A and B) BALB/c mice bearing subcutaneous tumours and images of tumour xenografts are shown after inoculation of HepG2/OXA‐shUCA1 cells or HepG2/OXA‐shNC cells followed by treatment with OXA or normal saline (NS) (10 mg/kg, i.p., once a week for 4 weeks). (C and D) Tumor size and weight of HepG2/OXA shUCA1 xenografts or control counterparts with either OXA or saline treatment were observed and recorded. Data indicates mean ± SD. N = 5, but one mouse died with an undetermined cause after administrated with OXA for 1wk in shUCA1‐transfected HepG2/OXA group. *p < 0.05, **p < 0.01, ***p < 0.001. E, Expression of UCA1 in tumor xenografts was measured using qRT‐PCR. Bars are mean ± SD from 3 independent experiments. **p < 0.01, compared NC group. F‐G, Western blot analysis demonstrated that the expression of p‐AKT and p‐mTOR is suppressed in the HepG2/OXA cells after transfection with shUCA1. Bars are mean ± SD from 3 independent experiments. *p < 0.05, **p < 0.01, compared to NC groups

Combining the above results in clinical specimens and in vitro, we speculated that knockdown of UCA1 may re‐sensitize HCC cells to OXA in vivo. Thereby, HepG2/OXA‐shUCA1 cells or HepG2/OXA‐shNC cells were subcutaneously injected into the flanks of BALB/c mice separately. As the results shown in Figure 4A‐E, knockdown of UCA1 in HepG2 cells could dramatically inhibit tumor xenograft formation than those of control HepG2/OXA‐shNC group with saline treatment. Furthermore, administration of OXA (10 mg/kg, i.p., once a week for 4 weeks) triggered more dramatic regression of HepG2/OXA‐shUCA1 tumor xenografts than HepG2/OXA‐shNC xenografts, compared with saline groups. Consistent with the results in vitro, the expression of p‐AKT and p‐mTOR was decreased in HepG2/OXA‐shUCA1 samples compared with those of HepG2/OXA‐shNC groups (*p *<* *0.05 or *p *<* *0.01, Figure 4F,G). The total amount of AKT and TOR was not significantly affected in both groups. All these data suggest that UCA1 knockdown re‐sensitizes HCC to OXA therapy partially via activation of AKT/mTOR pathway in vivo.

## 4 DISCUSSION

HCC is notoriously refractory cancer which is resistant to conventional chemotherapy and radiotherapy, carrying a dismal prognosis. LncRNAs are emerging as new players in the cancer paradigm demonstrating potential roles in both oncogenic and tumor‐suppressive pathways.^45^ In recent years, accumulating evidence indicates that lncRNAs exert a pivotal role in the invasion, metastasis, and chemoresistance of HCC.^46^, ^47^ Recently, some lncRNAs have been shown to function as chemoresistance inducers in HCCs, such as UCDR, H19, and MALAT‐1.^48^, ^49^ Moreover, researchers have demonstrated that UCA1 acts as a driver of tumor progression and drug resistance in many cancers. For example, Cheng et al found that the increased UCA1 expression in EGFR‐TKI‐resistant NSCLC cells results in hyperactivation of AKT/mTOR signaling pathway, whereas downregulation of UCA1 in these cells partially restore sensitivity to EGFR‐TKI.^30^ Similarly, UCA1 increases the cisplatin resistance of bladder cancer cells by enhancing Wnt6 expression, and thus represents a potential target to overcome chemoresistance in bladder cancer.^50^ Besides, UCA1 also promotes breast cancer cells to resist tamoxifen by activating AKT/mTOR axis.^48^ As reported by J Wang et al, ovarian cancer cells acquire paclitaxel resistance due to the excessive activation of MDR1 which is under the positive regulation of UCA1/miR‐129.^51^ Besides, exosomal UCA1 is a critical mediator of resistance to gefitinib in NSCLC which enhance FOSL2 expression by repressing miR‐143.^52^

In this study, we performed a retrospective study of 75 clinical samples and found that UCA1 is greatly upregulated in OXA‐resistant HCC clinical samples. Furthermore, we found that the high expression of UCA1 is positively correlated with serum AFP, tumor size, and distant metastasis, and predicts poor prognosis. Consistently, Xiao et al reported that high UCA1 expression in HCC samples is positively associated with tumor size, vascular invasion, and American Joint Committee on Cancer (AJCC) stage in a retrospective study of 60 pairs of clinical cases.^53^ Wang et al detected UCA1 expression in 98 pairs of human HCC and corresponding nontumourous liver specimens, and identified that UCA1 is aberrantly upregulated in HCC tissues, and correlated with TNM stage, metastasis, and postoperative survival.^19^ But they did not found any significant correlation between UCA1 expression and serum AFP level or tumor size. Moreover, our data showed that UCA1 is not an independent prognostic factor for OS in HCC patients. Inconsistent with our results, Wang et al indicated that UCA1 is an independent prognostic factor for survival in HCC patients. This difference might be caused by the relatively small sample size and more samples remain to be further analyzed.

According to previous reports, UCA1 induces chemo‐resistance mainly through the activation of cellular signaling pathways which confers cancer cells’ abilities to adapt to harsh environments with chemo‐reagents. Among these pathways, AKT/mTOR axis is closely associated with UCA1 function. More importantly, AKT/mTOR axis is a predominant signaling pathway in chemo‐resistance of HCC. For instance, Qian et al illustrated that a concomitant suppression of MDR1 and MRP1 expression and activation of p‐AKT confer the multidrug resistance to ADM and 5‐FU in HCC.^54^ Besides, Zhou et al found that c‐MYC, a downstream transcriptional regulator of AKT, is upregulated when AKT is phosphorylated, therefore assisting HCC to resist cisplatin.^55^ On the other hand, activated AKT also suppresses the apoptotic initiator Bcl‐2, but elevates the expression of Bax when HCC cells acquire OXA‐resistance.^56^ Also, microRNA‐19a‐3p promotes tumor metastasis and sorafenib resistance through the PTEN/Akt pathway in HCC.^57^ Therefore, we dissected the molecular mechanism of UCA1 by focusing on detecting the activation level of AKT/mTOR pathway in our study, and found a positive correlation between AKT/mTOR activities and the OXA resistance in HCC. Additionally, OXA has been administrated for advanced HCC patients due to the safety and economic applicability,^12^ but it is challenged by the development of inevitable chemo‐resistance in patients. In this study, we established two in vitro OXA‐resistant models, HepG2/OXA and SMMC‐7721/OXA cells, with resistance index (RI) 6.34 and 2.13 to mimic the acquired resistance during OXA treatment in HCC patients. Then we validated that upregulation of UCA1 eliminates sensitivity of HCC to OXA through activating AKT/mTOR pathway in vitro and in vivo, whereas the response efficacy of OXA in HCC is restored after decreasing the expression of UCA1.

LncRNAs are known to activate signaling pathway by modulation of miRNAs expression. Many studies have reported that lncRNAs facilitate chemotherapy resistance by regulating miRNAs. For example, Qu et al demonstrated that high expression of lncARSR promotes renal cancer resistance to sunitinib by competitively binding Mir‐34 and Mir‐449 and activating the STAT3/AKT/ERK signaling pathway.^58^ And Lin W et al revealed that overexpressed LINC00473 promotes the taxol resistance via inhibition of tumor suppressor miR‐15a and then suppression of BCL‐2‐related antiapoptosis and upregulation of MDR signals in colorectal cancer.^59^ Additionally, UCA1 might function as an oncogene in tongue squamous cell carcinoma (TSCC) through regulating the miR‐138‐5p/ CC chemokine receptor (CCR7) axis, and the interaction between miR‐138‐5p and UCA1 or CCR7 has been identified.^60^ In this study, we found that miR‐138‐5p could bind to UCA1 and upregulation of miR‐138‐5p expression exerts the opposite effect on AKT/mTOR pathway activation induced by high UCA1 expression. Taken together, our results indicate that upregulated UCA1 contributes to OXA resistance in HCC via suppression of miR‐138‐5p expression and activation of AKT/mTOR pathway. These findings suggest that targeting UCA1/miR‐138‐5p/AKT/mTOR signaling axis potentially represents a novel therapeutic option for overcoming OXA resistance in HCC.

## 5 CONCLUSION

In summary, our study reports that UCA1 contributes to the OXA resistance of HCC in vitro and in vivo as well as in clinical patients. Mechanistically, UCA1 downregulates miR‐138‐5p, thereby leading to activation of AKT/mTOR signaling pathway in HCC cells. These findings illustrate a novel mechanism for understanding OXA resistance in HCC, and suggest that UCA1/miR‐138‐5p/AKT/mTOR signaling axis has a potential therapeutic value for the treatment of OXA resistance in HCC patients.

## CONFLICT OF INTEREST

None.

## AUTHOR'S CONTRIBUTIONS

Guolin Huang and Li Li performed main experiments, analyzed data, and drafted the manuscript. Chaoyong Liang collected and classified the human tissue samples. Cuifang Teng, Yingxing Pang, Jinjing Song, and Tongtong Wei performed some experiments. Hanlin Wang analyzed some data. Xiaoli Liao and Yongqiang Li collected and tested clinical specimens, and followed up the patients. Jie Yang designed the study. Fei Yu performed supplementary experiments.

## Supporting information

Table S1

Table S2

Table S3

Table S4

## Data Availability

The data that supports the findings of this study are available on request from the corresponding author.
