# Supplementary material for: Upregulated UCA1 contributes to oxaliplatin resistance of hepatocellular carcinoma through inhibition of miR‐138‐5p and activation of AKT/mTOR signaling pathway
Source: Pharmacol Res Perspect. 2021 Feb 10;9(1):e00720. doi: 10.1002/prp2.720 (PMC7874507; doi:10.1002/prp2.720)
Supplement: Supplementary file 1 — Table S1 [file PRP2-9-e00720-s001.docx]

**Table S1 Primers and primer sequence**

| primers | primer sequence |
| --- | --- |
| UCA1 | forward 5′-ACCAATACTGGGCTCTGAGTGGCTAT-3′ |
|  | reverse 5′-ACAGCCACATTTGATTTTGCTTCAG-3′ |
| miR-138-5p | forward 5′-GCGAGCTGGTGTTGTGAATC-3′ |
|  | reverse 5′-AGTGCAGGGTCCGAGGTATT-3′ |
| miR-138-5p RT | 5′-GTCGTATCCAGTGCAGGGTCCGAGGTATTCGCACTGGATACGACCGGCCT-3′ |
| U6 | forward 5′-AGAGAAGATTAGCATGGCCCCTGC-3′ |
|  | reverse 5′-ATCCAGTGCAGGGTCCGAGG-3′ |
| U6 RT | 5′-GTCGTATCCAGTGCAGGGTCCGAGGTATTCGCACTGGATACGACAAAATA-3′ |
| GAPDH | forward 5′-AGCCACATCGCTCAGACA-3′ |
|  | reverse 5′-GCCCAATACGACCAAATCC-3′ |
