# Supplementary material for: Upregulated UCA1 contributes to oxaliplatin resistance of hepatocellular carcinoma through inhibition of miR‐138‐5p and activation of AKT/mTOR signaling pathway
Source: Pharmacol Res Perspect. 2021 Feb 10;9(1):e00720. doi: 10.1002/prp2.720 (PMC7874507; doi:10.1002/prp2.720)
Supplement: Supplementary file 2 — Table S2 [file PRP2-9-e00720-s002.docx]

**Table S2 Catalogue numbers and concentration of the antibodies**

| Antibody | Catalogue number | Concentration | Company |
| --- | --- | --- | --- |
| mTOR | 2983P | 1:1000 | CST, USA |
| p-mTOR | 5536P | 1:2000 | CST, USA |
| AKT | 4691P | 1:1000 | CST, USA |
| p-AKT | 4060P | 1:1000 | CST, USA |
| GADPH | 10494-1-AP | 1:10000 | Proteintech, China |
