# Supplementary material for: Upregulated UCA1 contributes to oxaliplatin resistance of hepatocellular carcinoma through inhibition of miR‐138‐5p and activation of AKT/mTOR signaling pathway
Source: Pharmacol Res Perspect. 2021 Feb 10;9(1):e00720. doi: 10.1002/prp2.720 (PMC7874507; doi:10.1002/prp2.720)
Supplement: Supplementary file 3 — Table S3 [file PRP2-9-e00720-s003.docx]

**Table S3 Relationship between UCA1 and clinicopathological characteristics of HCC patients**

| clinicopathological indices |  | No. of cases | UCA1 | | *P*-value |
| --- | --- | --- | --- | --- | --- |
|  |  |  | Low | High |  |
| Gender | Male | 69 | 28 | 41 | 1.000 |
|  | Female | 6 | 2 | 4 |  |
| Age | ≤45 | 29 | 12 | 17 | 0.968 |
|  | >45 | 46 | 18 | 26 |  |
| HBV infection | Absent | 10 | 2 | 8 | 0.298 |
|  | Present | 65 | 28 | 37 |  |
| Serum AFP | ≤400 | 40 | 19 | 18 | **0.048*** |
|  | >400 | 35 | 11 | 27 |  |
| Liver cirrhosis | Absent | 19 | 5 | 14 | 0.159 |
|  | Present | 56 | 25 | 31 |  |
| Child-Pugh classification | A | 45 | 20 | 25 | 0.471 |
|  | B | 12 | 3 | 9 |  |
|  | C | 18 | 7 | 11 |  |
| Tumor size(cm) | ≤5 | 13 | 11 | 6 | **0.018*** |
|  | >5 | 62 | 19 | 39 |  |
| Tumor number | Single | 32 | 10 | 22 | 0.182 |
|  | Multiple | 43 | 20 | 23 |  |
| Vascular invasion | Absent | 27 | 9 | 18 | 0.377 |
|  | Present | 48 | 21 | 27 |  |
| Distant metastasis | Absent | 61 | 28 | 33 | **0.021*** |
|  | Present | 14 | 2 | 13 |  |

Note. **P* < 0.05, chi square test (2-side).
