# Supplementary material for: Upregulated UCA1 contributes to oxaliplatin resistance of hepatocellular carcinoma through inhibition of miR‐138‐5p and activation of AKT/mTOR signaling pathway
Source: Pharmacol Res Perspect. 2021 Feb 10;9(1):e00720. doi: 10.1002/prp2.720 (PMC7874507; doi:10.1002/prp2.720)
Supplement: Supplementary file 4 — Table S4 [file PRP2-9-e00720-s004.docx]

**Table S4 Univariate and Multivariate analysis identifies factors influencing overall survival of HCC patients**

| Factors | Univariate analysis | | Multivariate analysis | |
| --- | --- | --- | --- | --- |
|  | HR (95% CI) | P-value | HR (95% CI) | P-value |
| Gender | 0.320 (0.074-1.394) | 0.129 |  |  |
| Age | 0.680 (0.318-1.452) | 0.319 |  |  |
| HBV infection | 11.570 (1.830-73.000) | 0.009* | 1.950 (0.639-5.948) | 0.241 |
| Serum AFP | 1.154 (0.553-2.411) | 0.702 |  |  |
| Liver cirrhosis | 1.059 (0.470-2.384) | 0.891 |  |  |
| Child-Pugh classification B | 1.223 (0.508-2.943) | 0.653 |  |  |
| Child-Pugh classification C | 2.324 (1.151-4.695) | 0.019* | 0.493 (0.196-1.242) | 0.134 |
| Tumor size (cm) | 0.572 (0.270-1.212) | 0.145 |  |  |
| Tumor number | 1.659 (0.774-3.555) | 0.193 |  |  |
| Vascular invasion | 1.480 (0.689-3.184) | 0.315 |  |  |
| Distant metastasis | 0.404 (0.099-1.634) | 0.203 |  |  |
| UCA1 | 2.104 (1.118-3.961) | 0.021* | 1.376 (0.700-2.706) | 0.354 |

Note. HR, Hazard ratio. CI, confidence interval. **P*<0.05

 The univariate analysis and multivariate analysis were conducted to evaluate which index could potentially be prognostic factors for overall survival (OS) in HCC patients. As illustrated in Table S4, HCC patients with HBV infection, Child-Pugh classification C and high expression of UCA1 have a shorter OS based on the univariate log-rank analysis. Meanwhile, multivariate cox proportional hazards analysis revealed that none of three factors is an independent prognostic factor for OS in HCC patients.
